# Genetic Diversity Evaluation of 70 Chewing Cane Germplasm Resources Based on Phenotypic Traits

**DOI:** 10.3390/plants14193111

**Published:** 2025-10-09

**Authors:** Jing Xie, Zhuang Liu, Hailong Chang, Chuiming Zhang, Qinggan Liang, Zhuqing Wang, Yinjie Cheng, Qinnan Wang, Jiantao Wu

**Affiliations:** Guangdong Sugarcane Genetic Improvement Engineering Center, Institute of Nanfan & Seed Industry, Guangdong Academy of Sciences, Guangzhou 510316, China

**Keywords:** genetic similarity, genetic correlation, genetic resources, cluster analysis, private allele

## Abstract

Chewing cane is primarily consumed as fresh fruit and contains a variety of essential amino acids and iron. The identification and evaluation of chewing cane germplasm resources are prerequisites for the rational utilization of these resources, and phenotypic traits provide a direct and convenient means to determine breeding objectives. To facilitate the utilization and development of chewing cane germplasm, thirty-six phenotypic traits in 70 chewing cane germplasm resources were analyzed. The results revealed rich genetic diversity among the chewing cane resources. Seven phenotypic traits exhibited relatively high diversity and considerable potential for genetic improvement. The three groups exhibited relatively small genetic distances and relatively high genetic similarity coefficients, among which the Local varieties (Lv) group displayed the highest genetic diversity indices and retained more private alleles. Based on genetic similarity, the resources were classified into 3 groups, 5 subgroups, and 5 subclasses through cluster analysis, with more than half (54%) of the chewing cane materials forming a separate cluster. A total of 26 private alleles associated with 13 different phenotypic traits in chewing cane were identified across the three groups. The results of genetic diversity analysis based on phenotypic data provide important references for the utilization of chewing cane germplasm resources, selection of parents, and variety breeding.

## 1. Introduction

Chewing cane (*Saccharum* spp.), also known as the chewing type of sugarcane, is cultivated exclusively for fresh consumption. It is characterized by thin and crispy nodes that are easy to peel; low fiber content; moderate sugar content; abundant and clear sweet juice; and a crisp, refreshing texture. It is rich in protein and essential trace elements such as iron, calcium, phosphorus, manganese, and zinc [1]. Chewing cane has the effects of quenching thirst and providing cooling and detoxification benefits. Moreover, it enjoys a long market period, a large sales volume, and a relatively low price, making it one of the most popular fresh foods among consumers [2,3,4,5]. In China, chewing cane is mainly cultivated in Guangdong, Guangxi, Yunnan, Fujian, and Hainan provinces and is an important characteristic economic crop in southern China. Its cultivation history in China spans at least 2000 years [6,7]. In recent years, the demand for chewing cane has been continuously increasing, accompanied by an expansion of planting areas. It is now cultivated in 15 provinces and regions, with an annual planting area of approximately 250,000 hectares and a production volume of about 350 million tons [8,9,10].

The study of germplasm resources forms the foundation of crop breeding. Research on biological genetic diversity, widely applied in genetics, breeding, and taxonomy, is valued for its simplicity, ease of operation, and efficiency [11,12]. Germplasm can be evaluated using genetic diversity indices and coefficients of variation based on morphological and agronomic traits, thereby revealing the diversity characteristics of different germplasm resources and providing a basis for their utilization and development [13]. With advancements in breeding techniques, chewing cane germplasm resources have been continuously introduced and selected. The scientific and efficient screening of superior sugarcane materials has become an important task in breeding programs [14,15,16,17]. Agronomic and yield traits objectively reflect the comprehensive characteristics of crop varieties, and their phenotypes can be directly assessed using appropriate tools. Consequently, these traits are frequently employed by breeders as key indicators for evaluating and identifying germplasm resources [18,19,20]. Integrating multiple desirable agronomic traits into a single genotype is an essential goal in sugarcane breeding. Selecting parents with superior agronomic traits and designing suitable hybrid combinations constitute important strategies for improving sugarcane varieties [21,22,23]. Genetic diversity serves as a critical measure of a species’ capacity to adapt to environmental changes and provides a theoretical foundation for the effective utilization of crop germplasm resources [24,25,26].

At present, a relatively small number of researchers have used molecular markers to evaluate the genetic diversity of chewing cane germplasm resources [27,28,29]. However, studies on the genetic diversity of chewing cane based on phenotypic data are still rare. Prolonged lack of systematic investigation has led to the disorder and degeneration of local chewing cane varieties, with little varietal renewal. Furthermore, inadequate attention to cultivation management has resulted in severe disease incidence, deterioration in quality, reduced storage ability, and yield decline [8,30]. There is an urgent need to develop new chewing cane varieties that are high-quality, high-yielding, and disease-resistant. China possesses numerous excellent local chewing cane varieties, such as Luohanzhe, Datian Xuezhe, and Guangzhou Qingpi, which have been cultivated for over 50 years and still retain superior traits and high productivity. Therefore, it is essential to investigate, characterize, and systematically evaluate these local varieties while exploring chewing cane germplasm resources across different regions of China. In the present study, field-collected phenotypic data were used to assess the genetic diversity and kinship of chewing cane germplasm resources from various origins, with the aim of identifying superior germplasm for introduction and breeding in diverse planting areas.

## 2. Results

### 2.1. Analysis of Genetic Diversity of Qualitative Traits

The diversity index of qualitative traits reflects the distribution of traits at different levels. A genetic diversity analysis was conducted on 27 qualitative traits of 70 chewing cane germplasm resources (Table 1). A total of 82 types of variations were detected, with each trait exhibiting between 2 and 6 types of variations. On average, each trait had 2.83 types of variations. Considerable variation was observed, with *CV* ranging from 21.93% to 54.21%. Among the 27 traits, BFo exhibited the greatest genetic variation, while LC showed the least genetic variation.

ICE showed the highest genetic diversity index, with the highest proportion (31%) in grade 3. On the other hand, 57HG showed the lowest genetic diversity index, with the highest proportion (94%) in grade 1. The Shannon’s information index (*I*) values of ICU, BS, LSC, IWP, BFo, IF, and ICE were all greater than 1, and their variation ranges were also relatively large. The genetic diversity index of stalk-related traits was the highest, with average values of 1.173 (*I*) and 0.610 (*h*), respectively. Next were leaf-related traits, with average values of 0.776 (*I*) and 0.462 (*h*), respectively. Finally, the genetic diversity index of bud-related traits was the lowest, with average values of 0.655 (*I*) and 0.392 (*h*), respectively. These findings indicate that the diversity levels of these qualitative traits were comparatively high, making them important reference indicators for evaluating the genetic diversity of chewing cane germplasm resources.

The frequency distribution of different grades of phenotypic traits of chewing cane germplasm resources in three sources was analyzed (Table 1). The most common grades for the stalk-related traits (IF, IA, WC, CP, CC, WB, Pip, Pit, GBF, GBCU and GBCE) were grade 1 or 2. For the bud-related traits (BFu, BP, 10HG, BS, BWS and LB), the highest frequency occurred at grade 1 or 2. For the leaf-related traits (LP, LC, LSP and 57HG), the highest frequency occurred at grade 1 or 2. The distribution frequencies of the six traits (AR, ICU, ICE, IWP, BFo and LSC) were more dispersed, not concentrated in a single grade.

### 2.2. Analysis of Genetic Diversity of Quantitative Traits

Statistical analyses were conducted on the mean, standard deviation (*SD*), coefficient of variation (*CV*), and genetic diversity index (*I* and *h*) for 9 quantitative traits (PH, SD, WpP, CY, Br, SucC, SugC, SL and IN) of the 70 tested germplasm resources (Table 2). *CV* values ranged from 11.97% to 43.82%, with a mean of 22.92%, indicating significant phenotypic variations among 70 different materials. SugC exhibited the highest *CV*, indicating substantial dispersion, followed by WpP and CY. The remaining six traits showed lower *CV* values, indicating a smaller potential for genetic improvement. SugC recorded the highest values for both indices, followed by Br, SucC, and SD.

**Figure 1:**
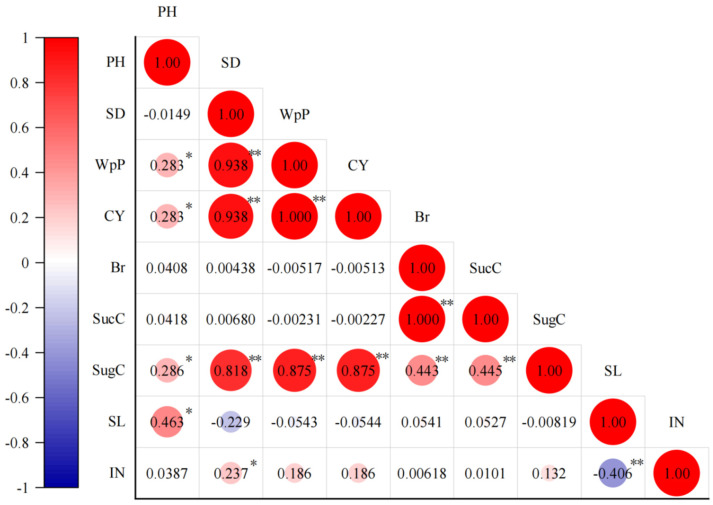
Correlation analysis of quantitative traits of chewing cane germplasm resources. * and ** represented significant and extremely significant correlations, respectively. Larger circles and darker colors indicated stronger correlations. Nine quantitative traits were Plant height (PH, cm), Stalk diameter (SD, cm), Weight per plant (WpP, kg), Cane yield (CY, t/hm^2^), Brix (Br, %), Sucrose content (SucC, %), Sugar content (SugC, t/hm^2^), Stem length (SL, cm), and Internode number (IN, cm).

Further analysis of the bivariate correlations among the nine quantitative traits is presented in Figure 1. PH was significantly positively correlated with WpP, CY, and SugC, and extremely significantly positively correlated with SL. SD was significantly positively correlated with WpP, CY, and SugC, and also positively correlated with IN. WpP was significantly positively correlated with both CY and SugC. CY showed a significant positive correlation with SugC and an insignificant negative correlation with Br, SucC, and SL. Br displayed highly significant positive correlations with both SucC and SugC. SucC and SugC were also highly significantly positively correlated. SL and IN showed a highly significant negative correlation.

### 2.3. Genetic Diversity and Correlation of Chewing Cane Germplasm Resources Across Three Populatons

Based on the different frequency distributions of 36 phenotypic traits, genetic diversity analysis was conducted on three different origins of chewing cane germplasm resources (Table 3). The highest *I* and *h* values were recorded in the Lv group, with values of 1.083 and 0.574, respectively, followed by the Bv group. The Iv group exhibited lower values than the overall population average. The F-statistics analysis revealed a genetic differentiation coefficient of 0.72%, indicating that 0.72% of the genetic variation occurred among origins, while 99.28% occurred within origins.

The genetic distances among the three sugarcane populations were relatively small, and genetic similarity was high (Table 4). In particular, the genetic similarity coefficients between Bv and Iv, and between Bv and Lv, were relatively high, while the corresponding genetic distances were relatively small. This may reflect the fact that Bv materials were developed through crossbreeding and selection involving germplasm materials from both Iv and Lv.

### 2.4. Cluster Analysis and PCoA of Chewing Cane Germplasm Resources

**Figure 2:**
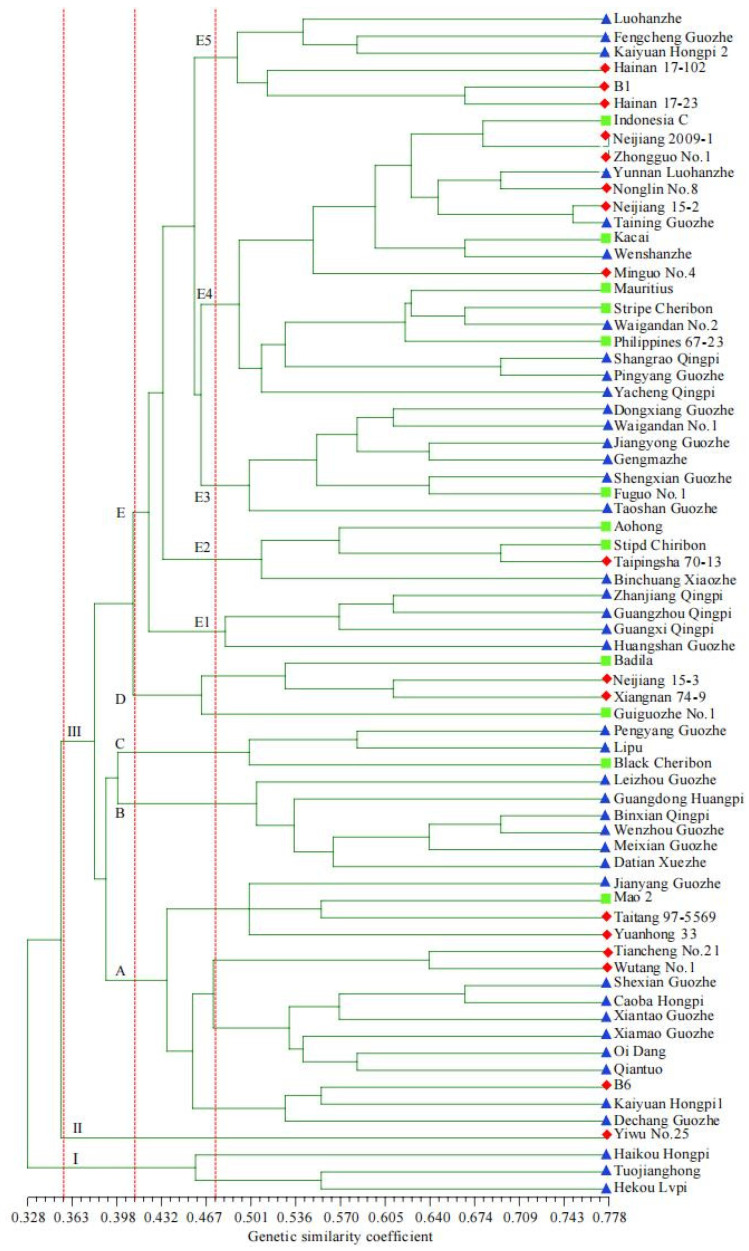
Cluster dendrogram of 70 chewing cane germplasm resources based on genetic similarity. Genetic similarity was calculated using the sm coefficient. The red rhombus, green square and blue triangle represented Bred variety, Introduced variety and Local variety, respectively. Seventy chewing cane resources were divided into three groups (I, II, and III) at the threshold of 0.361. Group III was further divided into five subgroups (A, B, C, D and E) at the threshold of 0.412. Subgroup E was further divided into five subclasses (E1–E5) at the threshold of 0.474.

The genetic distances among the 70 chewing cane germplasm materials ranged from 0.022 to 0.508, with an average of 0.116 (Table S3). The smallest genetic distance was observed between Nonglin No. 8 and Tiancheng No. 21, while the largest distance occurred between Kaiyuan Hongpi 1 and Zhanjiang Qingpi. This was followed by distances of 0.497 between Kaiyuan Hongpi 1 and Jianyang Guozhe, and 0.483 between Pingyang Guozhe and Jianyang Guozhe. A cluster tree was constructed, dividing 70 samples of sugarcane germplasm into three different groups (I, II, and III) based on a similarity coefficient threshold of 0.361 (Figure 2). A two-way Mantel test was conducted to verify the accuracy of the phenotypic data used to distinguish the chewing sugarcane materials. The results showed that there was a highly significant correlation among the common eigenvalues obtained from the cluster analysis (r = 0.61194, *p* = 0.0020).

Group I included three chewing cane accessions—Haikou Hongpi, Hekou Lvpi, and Tuojianghong—from Lv. The majority of the materials in this group possess the following characteristics: AR-none, IF-cylindrica, IA-upright pattern, ICU and ICE-yellow or green, WC-none, CP and CC-none, WB-obvious, IWP-middle, Pip and Pit-middle, GBF-expansion, GBCU and GBCE-grey orange, BFo-elliptic, BFu-shallow, BP-reach, 10HG-none, BS-middle, BWS-wide, LB-none, LP-erect with carve near tip, LSC-yellow green, LSP-easily detachable, LSC-green with purple spots, and 57HG-none. The average values of the nine quantitative traits—PH, SD, WpP, CY, Br, SucC, SugC, SL, and IN—were: 338.3 cm, 2.93 cm, 2.288 kg, 154.468 t·hm^−2^, 18.8%, 12.6%, 19.3 t·hm^−2^, 9.9 cm, and 30, respectively (Table S4).

Group II contained a single accession, Yiwu No. 25, from Bv. This material was characterized by: AR-less, IF-cylindrical, IA-zigzag pattern, ICU and ICE-purple, WC-none, CP and CC-none, WB-obvious, IWP-thick, Pip and Pit-none, GBF-expansion, GBCU-yellow green, GBCE-green, BFo-roundness, BFo-shallow, BP-reach, 10HG-none, BS-middle, BWS-wide, LB-none, LP-erect, LC-green, LSP-easily detachable, LSC-purple, and 57HG-thick. The values of the nine quantitative traits—PH, SD, WpP, CY, Br, SucC, SugC, SL, and IN—were: 315.0 cm, 3.20 cm, 2.533 kg, 171.004 t·hm^−2^, 18.8%, 12.6%, 21.6 t·hm^−2^, 11.6 cm, and 29, respectively (Table S4).

Based on the similarity coefficient threshold of 0.412, Group III was further divided into five subgroups (A, B, C, D and E). Subgroup A comprised fifteen materials—B6, Caoba Hongpi, Dechang Guozhe, Jianyang Guozhe, Kaiyuan Hongpi1, Mao2, Oi Dang, Qiantuo, Shexian Guozhe, Taitang 97-5569, Tiancheng No.21, Wutang No.1, Xiamao Guozhe, Xiantao Guozhe, and Yuanhong 33. These accessions were characterized by: AR-less, IF-cylindrical, IA-zigzag-pattern, ICU and ICE-yellow or green, WC-have, CP and CC-have, GBF-expansion, IWP-middle, Pip and Pit-none, GBF-unexpanded, GBCU-yellow green, GBCE-grey orange, BFo-elliptic, BFu-none; BP-reach, 10HG-none, BS-small, BWS-narrow, LB-none, LP-erect with carved near tip, LC-green, LSP-easily detachable, LC-green, and 57HG-none. The mean values of the nine quantitative traits—PH, SD, WpP, CY, Br, SucC, SugC, SL, and IN—were 340.9 cm, 3.04 cm, 2.634 kg, 177.776 t·hm^−2^, 19.8%, 13.7%, 24.3 t·hm^−2^, 13.5 cm, and 29, respectively (Table S4).

Subgroup B comprised six chewing cane germplasm materials—Binxian Qingpi, Datian Xuezhe, Guangdong Huangpi, Leizhou Guozhe, Meixian Guozhe, and Wenzhou Guozhe. These accessions exhibited: AR-more, IF-drum, IA-upright pattern, ICU and ICE-yellow, WC-none, CP-have, CC-none, WB-obvious, IWP-middle, Pip and Pit-none, GBF-unexpansion, GBCU and GBCE-yellow green, BFo-elliptic, BFu-shallow, BP-high, 10HG-none, BS-big, BWS-narrow, LB-none, LP-erect with carved tip; LC-yellow green, LSP-easy detach, LSC-green, and 57HG-none. The mean values of the nine quantitative traits in Subgroup B were 296.7 cm, 2.95 cm, 2.041 kg, 137.800 t·hm^−2^, 21.2%, 15.2%, 21.0 t·hm^−2^, 11.5 cm, and 32, respectively (Table S4).

Subgroup C comprised three chewing cane accessions—Black Cheribon, Lipu, and Pengyang Guozhe. These materials were characterized by: AR-more, IF-cylindrical, IA-upright pattern, ICU-green, ICE-yellow, WC-none, CP and CC-none, WB-obvious, IWP-thin, Pip-middle, Pit-mild, GBF-unexpansion, GBCU-yellow green, GBCE-grey orange, BFo-oval, BFu-none, BP-reach, 10HG-none, BS-middle, BWS-narrow, LB-none, LP-drooping, LC-green, LSP-easily detachable, LC-green, and 57HG-none. The mean values of the nine quantitative traits in Subgroup C were 313.2 cm, 2.45 cm, 1.476 kg, 99.657 t·hm^−2^, 20.9%, 15.0%, 14.9 t·hm^−2^, 11.7 cm, and 31, respectively (Table S4).

Subgroup D comprised four chewing cane accessions—Badila, Guiguozhe No.1, Neijiang 15-3, and Xiangnan 74-9. These materials exhibited: AR-less, IF-drum, IA-zigzag pattern, ICU and ICE-green, WC-have, CP and CC-have, WB-not obvious, IWP-thin, Pip and Pit-none, GBF-expansion, GBCU-green, GBCE-grey orange, BFo-triangular, BFu-none, BP-reach, 10HG-none, BS-middle, BW-arrow, LB-have, LP-drooping, LC-green, LSP-easily detachable, LSC-green with purple spots, and 57HG-sparse. The mean values of the nine quantitative traits in Subgroup D were 291.5 cm, 3.10 cm, 2.265 kg, 152.860 t·hm^−2^, 19.8%, 13.7%, 21.0 t·hm^−2^, 11.9 cm, and 32, respectively (Table S4).

Subgroup E was the largest subgroup, consisting of 38 samples, including 9 Bv samples, 8 Iv samples, and 21 Lv samples. Among these five subgroups, subgroup A formed one branch, while subgroups B and C formed another branch, and subgroups D and E formed the third branch. Using a genetic similarity coefficient of 0.474 as the threshold, subgroup E was further divided into five subclasses. Subclass E1 included Guangxi Qingpi, Guangzhou Qingpi, Huangshan Guozhe, and Zhanjiang Qingpi. Subclass E2 comprised Aohong, Binchuang Xiaozhe, Stipd Chiribon, and Taipingsha 70-13. Subclass E3 contained Dongxiang Guozhe, Fuguo No.1, Gengmazhe, Jiangyong Guozhe, Shengxian Guozhe, Taoshan Guozhe, and Waigandan No.2. Subclass E4 included 17 accessions, such as Indonesia C, Kacai, Mauritius, and Minguo No.4. Subclass E5 comprised B1, Fengcheng Guozhe, Hainan 17-102, Hainan 17-23, Kaiyuan Hongpi2, and Luohanzhe. In terms of genetic differentiation, Subgroup E first diverged into Subclass E1, followed by Subclass E2, then Subclass E5, and finally into subclasses E3 and E4. Subclasses E3 and E4 formed a branch, indicating the closest genetic relationship between them.

**Figure 3:**
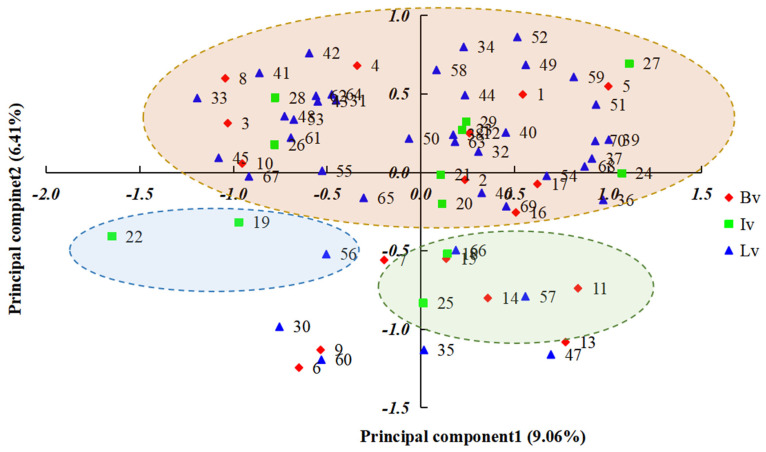
Principal component analysis (PCoA) of chewing cane germplasm resources. The red rhombus, green square and blue triangle represented Bred variety, Introduced variety and Local variety, respectively.

PCoA was conducted on 70 chewing cane germplasm resources from three different sources (Figure 3). The first, second, and third principal coordinates accounted for 9.06%, 6.14%, and 5.49% of the variance, respectively. Overall, the chewing cane germplasm resources were distributed across all quadrants. Most materials were concentrated in the upper part of the plot, with distributions among sources relatively scattered and intermingled. This pattern indicates a complex genetic background, with evidence of both gene flow and genetic differentiation among sources. Significant differences existed between individuals and the overall population. The three accessions of Group I clustered in the lower-left quadrant, Group II was located in the lower-right quadrant, and most materials in Group III were concentrated in the upper region. These results were consistent with those obtained from the cluster analysis.

These research results indicate that these 70 chewing cane germplasm resources can be classified into three groups, five subgroups and five subclasses based on the genetic similarity coefficient determined by phenotypic characteristics. It was worth noting that more than half (54%) of the materials were concentrated in a single subgroup.

### 2.5. Identification of Private Alleles in Chewing Cane Germplasm Resources

Based on the frequency of phenotypic trait grades among populations, private alleles in chewing cane germplasm resources were identified and enumerated (Table S5). A total of 26 private alleles associated with 13 phenotypic traits—10HG, Br, CY, GBCU, IF, IN, LC, PH, Pit, SD, SL, SucC, and WpP—were detected across three groups. Thirty-eight chewing cane accessions were found to contain at least one private allele, including seven, seven, and twenty-four accessions derived from Bv, Iv, and Lv, respectively (Table 5). Among these, 27 accessions carried a single private allele, six carried two private alleles, and five retained three private alleles. Accessions retaining private alleles may exhibit unique phenotypic traits and represent important genetic resources for sugarcane breeding.

## 3. Discussion

China has a long history of sugarcane cultivation and possesses a rich diversity of local sugarcane varieties, which are widely distributed. Many high-quality local chewing cane varieties are still cultivated in different regions, such as Guangzhou Qingpi and Leizhou Guozhe in Guangdong Province, Guangxi Qingpi and Lipu in Guangxi, Datian Xuezhe and Jianyang Guozhe in Fujian, and Wenshan Guozhe and Luohanzhe in Yunnan. During the course of sugarcane cultivation, these local varieties replaced Zhuzhe and Luzhe, which had historically played an important role in production [2,7,10].

In the 1930s, China introduced the *Saccharum officinarum* L. variety Badila from the Philippines. This variety is characterized by large stems, abundant juice, crisp texture, and a distinctive purplish-black color when exposed to sunlight. Due to its excellent commercial qualities, it was well-received by both producers and consumers and gradually became the dominant chewing cane variety cultivated in China [6]. However, despite their long cultivation history, local chewing cane varieties have often been grown using outdated agricultural practices, leading to significant loss of genetic purity. Some varieties are now on the verge of disappearance. The identification and preservation of these superior varieties are of considerable academic significance and practical value for enriching China’s chewing cane genetic resources and for further breeding and utilization [31]. In recent years, to meet market demands, researchers have collected, evaluated, and utilized existing chewing cane resources, selecting new multi-purpose varieties such as Xitian No. 21, Yiwu No. 25, and Guiguozhe No. 1 [10,32]. These new varieties have been widely adopted in certain regions, contributing significantly to the development of China’s sugarcane industry. In order to optimize the utilization effects of local varieties, introduced varieties and bred varieties, genetic diversity analysis was conducted on 36 phenotypic traits of 70 chewing sugar cane germplasm resources.

Among these three trait categories, the genetic diversity of stalk traits was the highest, followed by leaf traits, and the genetic diversity of flower bud traits was the lowest. A previous study on the phenotypic diversity of 106 Yutang series sugarcane parents indicated that the diversity of leaf traits was the highest, followed by bud traits, while the diversity of stalk traits was the lowest [18]. Similarly, a genetic diversity analysis of 138 Yacheng series parental lines revealed that the diversity of leaf traits was the highest, followed by stem traits, while the diversity of flower bud traits was the lowest [20]. Furthermore, an investigation into the genetic diversity of 18 qualitative traits in 42 local chewing cane germplasm resources [13] revealed that the traits with the highest diversity were internode color after exposure and bud shape, followed by internode shape, whereas stem shape exhibited the lowest diversity. The substantial ecological differences among sugarcane germplasm resources, combined with long-term cultivation and selection, have resulted in distinct phenotypic traits and corresponding differences in genetic diversity indices.

Due to the similarity in climate and environmental conditions in the regions of origin, as well as the fact that some parental lines were identical or closely related, the genetic background differences among the three groups were not substantial. Consequently, they exhibited relatively small genetic distances and high genetic similarity coefficients. Nevertheless, chewing cane genetic resources from different sources displayed distinct genetic characteristics. The genetic diversity indices of the Lv group were higher than those of the other two groups. Furthermore, the Lv group retained a greater number of private alleles (24), indicating higher genetic uniqueness. These private alleles may have resulted from a longer process of natural selection and adaptation to specific environmental conditions in local populations, thus possessing significant conservation value [31]. In contrast, the Bv and Iv groups may have lost certain advantageous alleles due to artificial selection and the limited founder effect. Future chewing cane breeding programs should therefore strengthen the conservation and breeding utilization of these local resources to preserve their unique genetic attributes [29].

The genetic similarity dendrogram indicated that geographical distance among the three groups had no significant effect on genetic differentiation and was not significantly correlated with genetic distance. Seventy samples can be classified into three major groups, five subgroups and five subclasses based on the genetic similarity coefficient determined by phenotypic characteristics. The results of a two-way Mantel test indicated that there was a highly significant correlation among the co-similarity values obtained from the cluster analysis [20]. The results of PCoA were consistent with those of the clustering analysis. Group I comprised three accessions (Haikou Hongpi, Hekou Lvpi, and Tuojianghong) from Lv; Group II contained one accession (Yiwu No. 25) from Bv; and Group III was further divided into five subgroups (A, B, C, D and E) based on the similarity coefficient threshold of 0.412. It was worth noting that the germplasm materials from the same origin were not always classified into the same category, which indicated that there was significant genetic differentiation among the chewing cane accessions from the same source. These clustering results provide important reference information for understanding the genetic relationships among the chewing cane germplasm resources [33].

Different groups or subgroups exhibited distinct phenotypic characteristics. In actual breeding work, it is recommended to select parental materials from different clustering groups—especially those individuals with distant genetic relationships—for the purpose of hybrid breeding [34]. It is not feasible to determine parental combinations solely on the basis of obvious agronomic traits, such as stem color. From a molecular perspective, chewing cane varieties with different stem colors may be genetically similar, whereas varieties with the same stem color may possess considerable genetic differences. Therefore, in chewing cane hybrid breeding, factors such as origin, geographical distribution, economic traits, and genetic relationships should all be considered. Selecting materials with relatively large overall genetic differences is conducive to identifying superior parental combinations [17,20]. For example, Kaiyuan Hongpi 1 (subgroup A) and Zhanjiang Qingpi (Subclass E2), as well as Pingyang Guozhe (Subclass E4) and Jianyang Guozhe (subgroup A), exhibited large genetic distance coefficients and may represent optimal parental combinations.

Private alleles are an important indicator for assessing the genetic uniqueness of crop populations. By analyzing the private alleles of chewing cane accessions from different geographical origins, genetic differences can be identified, and germplasms with unique genetic compositions can be selected for targeted hybrid breeding [35,36]. Accessions retaining private alleles may exhibit distinctive phenotypic traits and represent valuable genetic resources for sugarcane breeding. For instance, eight chewing cane accessions—Fuguo No. 1, Qiantuo, Lipu, Binchuang Xiaozhe, Huangshan Guozhe, Oi Dang, Taining Guozhe, and Taoshan Guozhe—retained the private allele Pit-grade2. These alleles can be used to rapidly distinguish varieties with similar genetic backgrounds and to serve as markers for precise selection of target traits in early generations, thereby improving breeding efficiency. Germplasm containing private alleles may also harbor valuable genetic variations beneficial for crop improvement. Chewing cane Aohong possessed three private alleles (PH-grade1, IN-grade1, and LC-grade3), when used as a parent, could be crossed with other parental lines to facilitate the targeted selection of varieties with reduced plant height, shorter internodes, and green leaves.

## 4. Materials and Methods

### 4.1. Plant Materials

A total of 70 chewing cane germplasm resources were obtained from the sugarcane germplasm resource nursery (18°39′ N, 109°15′ E) at the Institute of Nanfan & Seed Industry, Guangdong Academy of Sciences. These comprised 41 Local varieties (Lv), 12 Introduced varieties (Iv), and 17 Bred varieties (Bv). Detailed information on the origins of the chewing cane materials is provided in Table S1. The planting soil was sandy loam. The annual average temperature ranges from 22 to 27 °C, the annual sunshine duration is 1750 to 2650 h, the sunshine rate is 50% to 60%, and the annual average rainfall is 1000 to 2600 mm. It belongs to the tropical monsoon maritime climate.

### 4.2. Field Testing and Trait Investigation

All experimental materials were cultivated during the 2023–2024 growing seasons, including one cycle of plant cane and one cycle of ratoon cane. Seedlings were germinated in a nursery in January 2023 and transplanted to experimental plots in March 2023. The plant cane was harvested in December 2023, after which ratoon cane growth commenced in 2024. Cane stems were planted in pots, two buds per pot, and managed under standard irrigation and fertilization regimes until maturity. In December 2023 and 2024, ten mature plants per germplasm accession were randomly selected for the evaluation of 36 phenotypic traits (Table S2) during both the plant cane and ratoon cane stages. Trait assessment and data recording followed the Specification and Data Standard of Sugarcane Germplasm Resources Description [37].

Of the 27 qualitative traits evaluated, 15 were stalk-related traits, 7 were bud-related traits and 5 leaf-related traits. Each trait was determined through direct field observation and comparative analysis. The remaining nine traits were quantitative: plant height (PH), stalk diameter (SD), weight per plant (WpP), cane yield (CY), Brix (Br), sucrose content (SucC), sugar content (SugC), stem length (SL), and internode number (IN). Data for these traits were obtained from statistical analyses of measurements collected over two consecutive years. All 36 phenotypic traits were used to assess the genetic diversity of the chewing cane germplasm.

### 4.3. Data Processing and Statistical Analysis

Quantitative trait values were collected from field experiments. Membership function values for quantitative traits were calculated using the fuzzy membership function method [18,20], according to the following formula: $\mu (\chi_{i})=\frac{\chi_{i}-\chi_{i}\min}{\chi_{i}\max-\chi_{i}\min}(i\;=\;1,\;2,\;3\cdots \cdots 9)$, where μ(χ*_i_*) is the membership function value of the *i*-th trait for a given chewing cane germplasm, χ*_i_* is the trait value, and χ*_i_*max and χ*_i_*min are the maximum and minimum values, respectively, of that trait among all resources. Each quantitative trait value was normalized to the interval [0, 1].

Relative frequencies of each grade or interval for phenotypic traits were used to compute genetic diversity indices. Standard statistical descriptors—average value, standard deviation (SD), minimum (min.), maximum (max.), and coefficient of variation (*CV*)—were calculated using WPS Office for Windows v12.1. The frequency distribution of trait categories, Shannon’s information index (*I*), and Nei’s genetic diversity index (*h*) were computed using GenAlEx version 6.503 [38].

Genetic similarity and genetic distance were calculated using the Nei72 coefficient and the sm coefficient, respectively, in NTSYS-pc version 2.11e [39]. Cluster analysis was performed using the Sequential Agglomerative Hierarchical Nested (SAHN) method and the Unweighted Pair Group Method with Arithmetic Mean (UPGMA) based on genetic similarity coefficients, implemented in NTSYS-pc version 2.11e [39]. Cophenetic correlation was evaluated using the Mantel test with two-way matrix comparison in NTSYS-pc version 2.11e [40,41]. F-statistics (Fst), principal coordinates analysis (PCoA), and identification of private alleles were also conducted using GenAlEx version 6.503 [38].

## 5. Conclusions

The chewing cane germplasm resources examined exhibited extensive phenotypic variation, with pronounced differences observed across traits. Traits such as ICU, BS, LSC, IWP, Bfo, IF, and ICE demonstrated relatively high diversity and a wide range of genetic variations. The genetic distance among the three groups was relatively small, with the Lv group exhibiting the highest genetic diversity indices. Seventy chewing cane samples were clustered into three distinct groups, with a highly significant cophenetic correlation, consistent with the PCoA results. Combinations such as Kaiyuan Hongpi 1 (subgroup A) and Zhanjiang Qingpi (Subclass E2), as well as Pingyang Guozhe (Subclass E4) and Jianyang Guozhe (subgroup A), were identified as potential parental pairs for hybridization. Eight accessions—Fuguo No. 1, Qiantuo, Lipu, Binchuang Xiaozhe, Huangshan Guozhe, Oi Dang, Taining Guozhe, and Taoshan Guozhe—retaining the private allele Pit-grade2 can serve as markers for the precise selection of target traits in early breeding generations. Aohong, possessing three private alleles (PH-grade1, IN-grade1, and LC-grade3), is a promising parent for the targeted breeding of varieties with reduced plant height, shorter internodes, and green leaves. These results provide important references for the breeding of chewing cane.

## Figures and Tables

**Table 1:** Genetic diversity index of the 27 qualitative traits of chewing cane. *CV*, *I* and *h* represented coefficient of variation, Shannon’s information index, and Nei’s genetic diversity index, respectively.

Type	Trait	Distribution Frequency of Different Grades						*CV* (%)	*I*	*h*
		1	2	3	4	5	6			
Stalk-related traits	Aerial root (AR)	0.30	0.34	0.36				39.59	0.992	0.597
	Internode form (IF)	0.19	0.54	0.14	0.04	0.03	0.06	53.09	1.184	0.622
	Internode arrangement (IA)	0.54	0.46					34.43	0.674	0.481
	Internode color unexposed (ICU)	0.04	0.40	0.44	0.11			28.36	1.014	0.604
	Internode color exposed (ICE)	0.26	0.23	0.31	0.20			44.20	1.301	0.711
	Water crack (WC)	0.71	0.29					35.39	0.618	0.429
	Cork patch (CP)	0.54	0.46					34.43	0.649	0.458
	Cork cracks (CC)	0.59	0.41					35.08	0.644	0.453
	Wax band (WB)	0.60	0.40					35.25	0.664	0.471
	Internode wax powder (IWP)	0.29	0.37	0.30	0.04			41.46	1.131	0.638
	Pipe (Pip)	0.73	0.13	0.14				51.77	0.674	0.368
	Pith (Pit)	0.79	0.11	0.10				49.40	0.497	0.288
	Growth bands form (GBF)	0.59	0.41					35.08	0.666	0.473
	Growth bands color unexposed (GBCU)	0.81	0.11	0.07				46.29	0.516	0.305
	Growth bands color exposed (GBCE)	0.04	0.50	0.46				23.91	0.812	0.528
	Mean							39.84	1.173	0.610
Bud-related traits	Bud form (BFo)	0.37	0.20	0.09	0.34			54.21	1.140	0.647
	Bud furrow (BFu)	0.64	0.23	0.13				48.27	0.791	0.476
	Bud placement (BP)	0.16	0.63	0.21				29.71	0.435	0.307
	No.10 hair group (10HG)	0.94	0.06					22.12	0.107	0.059
	Bud size (BS)	0.03	0.51	0.27	0.19			31.43	1.029	0.579
	Bud wing size (BWS)	0.30	0.53	0.17				36.30	0.847	0.539
	Lateral budding (LB)	0.93	0.07					24.21	0.237	0.138
	Mean							35.18	0.655	0.392
Leaf-related traits	Leaf posture (LP)	0.17	0.79	0.04				23.93	0.537	0.339
	Leaf color (LC)	0.17	0.81	0.01				21.93	0.507	0.299
	Leaf sheath phimosis (LSP)	0.56	0.36	0.09				42.73	0.890	0.548
	Leaf sheath color (LSC)	0.14	0.41	0.40	0.04			33.21	1.114	0.641
	No.57 hair group (57HG)	0.67	0.19	0.14				50.06	0.833	0.484
	Mean							34.37	0.776	0.462
Mean								37.18	0.756	0.460

**Table 2:** Genetic diversity index of the 9 quantitative traits of chewing cane. *SD*, Min. and Max. represented standard deviation, minimum and maximum, respectively. *CV*, *I* and *h* represented coefficient of variation, Shannon’s information index and Nei’s genetic diversity index, respectively.

Trait	Average Value	*SD*	Min.	Max.	*CV* (%)	*I*	*h*
Plant height (PH, cm)	319.5	38.9	236.3	417.5	12.17	1.590	0.733
Stalk diameter (SD, cm)	2.88	0.54	1.76	4.30	18.83	1.880	0.827
Weight per plant (WpP, kg)	2.162	0.866	0.737	5.228	40.08	1.769	0.799
Cane yield (CY, t/hm^2^)	145.931	58.487	49.759	352.886	40.08	1.769	0.799
Brix (Br, %)	19.5	2.3	12.1	23.4	11.97	1.890	0.828
Sucrose content (SucC, %)	13.5	2.5	5.4	17.6275	18.83	1.890	0.828
Sugar content (SugC, t/hm^2^)	19.6	8.5	4.8	46.1	43.52	1.891	0.827
Stem length (SL, cm)	12.4	2.7	7.6	20.0	21.45	1.786	0.803
Internode number (IN, cm)	30	5	13	42	16.80	1.659	0.766
Mean					24.86	1.792	0.801

**Table 3:** Genetic diversity index of chewing cane germplasm resources across different populations. *I*, *h* and Fst represented Shannon’s information index, Nei’s genetic diversity index and F-statistics, respectively.

Population	*I*	*h*	Fst
Bred variety (Bv)	1.022	0.543	
Introduced variety (Iv)	0.948	0.524	
Local variety (Lv)	1.083	0.574	
Mean	1.007	0.537	0.0072

**Table 4:** Genetic distances and genetic similarity coefficients among the three types of source materials. The data in the upper right corner of the table represented genetic distance, while the data in the lower left corner of the table represented a genetic similarity coefficient.

Population	Bred Variety (Bv)	Introduced Variety (Iv)	Local Variety (Lv)
Bred variety (Bv)	—	0.884	0.898
Introduced variety (Iv)	0.123	—	0.927
Local variety (Lv)	0.107	0.076	—

**Table 5:** Chewing cane germplasm resources with private alleles.

Chewing Cane	Population	No. of Private Allele	Private Allele
Black Cheribon	Introduced varieties (Iv)	1	10HG-grade2
Datian Xuezhe	Local variety (Lv)	1	10HG-grade2
Zhanjiang Qingpi	Local variety (Lv)	2	Br-grade1, SucC-grade1
Binxian Qingpi	Local variety (Lv)	1	GBCU-grade3
Guiguozhe No.1	Introduced varieties (Iv)	1	GBCU-grade3
Haikou Hongpi	Local variety (Lv)	1	GBCU-grade3
Tuojianghong	Local variety (Lv)	1	GBCU-grade3
Hekou lvpi	Local variety (Lv)	2	GBCU-grade3, 10HG-grade2
Xiangnan 74-9	Bred variety (Bv)	1	IF-grade5
Fuguo No.1	Introduced variety (Iv)	2	IF-grade5, Pit-grade2
Kaiyuan Hongpi 1	Local variety (Lv)	1	IN-grade2
Kacai	Introduced variety (Iv)	1	IN-grade3
Neijiang 15-2	Bred variety (Bv)	1	IN-grade4
Waigandan No.1	Local variety (Lv)	1	IN-grade4
Yuanhong 33	Bred variety (Bv)	1	IN-grade4
Badila	Introduced variety (Iv)	1	PH-grade1
Hainan 17-102	Bred variety (Bv)	1	PH-grade1
Aohong	Introduced variety (Iv)	3	PH-grade1, IN-grade1, LC-grade3
Gengmazhe	Local variety (Lv)	1	PH-grade6
Xiantao Guozhe	Local variety (Lv)	1	PH-grade6
Wenshanzhe	Local variety (Lv)	3	PH-grade6, Br-grade2, SucC-grade2
Qiantuo	Local variety (Lv)	3	PH-grade6, IN-grade4, Pit-grade2
Lipu	Local variety (Lv)	2	PH-grade6, Pit-grade2
Binchuang Xiaozhe	Local variety (Lv)	1	Pit-grade2
Huangshan Guozhe	Local variety (Lv)	1	Pit-grade2
Oi Dang	Local variety (Lv)	1	Pit-grade2
Taining Guozhe	Local variety (Lv)	1	Pit-grade2
Taoshan Guozhe	Local variety (Lv)	1	Pit-grade2
Jianyang Guozhe	Local variety (Lv)	3	SD-grade10, WpP-grade10, CY-grade10
Shangrao Qingpi	Local variety (Lv)	1	SL-grade1
Shengxian Guozhe	Local variety (Lv)	1	SL-grade1
Caoba Hongpi	Local variety (Lv)	1	SL-grade10
Pingyang Guozhe	Local variety (Lv)	2	SL-grade10, IN-grade4
Jiangyong Guozhe	Local variety (Lv)	1	SL-grade2
Nonglin No.8	Bred variety (Bv)	1	SL-grade9
Taitang 97-5569	Bred variety (Bv)	3	WpP-grade8, CY-grade8, IN-grade4
Mao 2	Introduced variety (Iv)	2	WpP-grade9, CY-grade9
Yiwu No.25	Bred variety (Bv)	1	10HG-grade2

## Data Availability

Data are contained within the article and Supplementary Materials.

## Supplementary Materials

The following supporting information can be downloaded at: https://www.mdpi.com/article/10.3390/plants14193111/s1, Table S1. The information of 70 chewing cane germplasm resources. Table S2. Grading criteria for 27 qualitative traits of chewing cane germplasm resources. Table S3. The genetic distance matrix of 70 chewing cane germplasm resources. Table S4. The characteristics of different groups of chewing cane germplasm resources. Table S5. Private alleles in chewing cane across different origins.
