# Supplementary material for: Genetic Diversity Evaluation of 70 Chewing Cane Germplasm Resources Based on Phenotypic Traits
Source: Plants (Basel). 2025 Oct 9;14(19):3111. doi: 10.3390/plants14193111 (PMC12526319; doi:10.3390/plants14193111)
Supplement: Supplementary file 1 [file plants-14-03111-s001.zip › Table S1. The information of 70 chewing cane germplasm resources.pdf]

Table S1. The information of 70 chewing cane germplasm resources

No.	Material	Origin
1	Aohong	Introduced variety (Iv)
2	B1	Bred variety (Bv)
3	B6	Bred variety (Bv)
4	Badila	Introduced variety (Iv)
5	Binchuang Xiaozhe	Local variety (Lv)
6	Binxian Qingpi	Local variety (Lv)
7	Black Cheribon	Introduced variety (Iv)
8	Caoba Hongpi	Local variety (Lv)
9	Datian Xuezhe	Local variety (Lv)
10	Dechang Guozhe	Local variety (Lv)
11	Dongxiang Guozhe	Local variety (Lv)
12	Fengcheng Guozhe	Local variety (Lv)
13	Fuguo No.1	Introduced variety (Iv)
14	Gengmazhe	Local variety (Lv)
15	Guangdong Huangpi	Local variety (Lv)
16	Guangxi Qingpi	Local variety (Lv)
17	Guangzhou Qingpi	Local variety (Lv)
18	Guiguozhe No.1	Introduced variety (Iv)
19	Haikou Hongpi	Local variety (Lv)
20	Hainan 17-102	Bred variety (Bv)
21	Hainan 17-23	Bred variety (Bv)
22	Hekou lvpi	Local variety (Lv)
23	Huangshan Guozhe	Local variety (Lv)
24	Indonesia C	Introduced variety (Iv)
25	Jiangyong Guozhe	Local variety (Lv)
26	Jianyang Guozhe	Local variety (Lv)
27	Kacai	Introduced variety (Iv)
28	Kaiyuan Hongpi1	Local variety (Lv)
29	Kaiyuan Hongpi2	Local variety (Lv)
30	Leizhou Guozhe	Local variety (Lv)
31	Lipu	Local variety (Lv)
32	Luohanzhe	Local variety (Lv)
33	Mao2	Introduced variety (Iv)
34	Mauritius	Introduced variety (Iv)
35	Meixian Guozhe	Local variety (Lv)
36	Minguo No.4	Bred variety (Bv)
37	Neijiang 15-2	Bred variety (Bv)
38	Neijiang 15-3	Bred variety (Bv)
39	Neijiang 2009-1	Bred variety (Bv)
40	Nonglin No.8	Bred variety (Bv)

41	Oi Dang	Local variety (Lv)
42	Pengyang Guozhe	Local variety (Lv)
43	Philippines 67-23	Introduced variety (Iv)
44	Pingyang Guozhe	Local variety (Lv)
45	Qiantuo	Local variety (Lv)
46	Shangrao Qingpi	Local variety (Lv)
47	Shengxian Guozhe	Local variety (Lv)
48	Shexian Guozhe	Local variety (Lv)
49	Stipd Chiribon	Introduced variety (Iv)
50	Stripe cheribon	Introduced variety (Iv)
51	Taining Guozhe	Local variety (Lv)
52	Taipingsha 70-13	Bred variety (Bv)
53	Taitang 97-5569	Bred variety (Bv)
54	Taoshan Guozhe	Local variety (Lv)
55	Tiancheng No.21	Bred variety (Bv)
56	Tuojianghong	Local variety (Lv)
57	Waigandan No.1	Local variety (Lv)
58	Waigandan No.2	Local variety (Lv)
59	Wenshanzhe	Local variety (Lv)
60	Wenzhou Guozhe	Local variety (Lv)
61	Wutang No.1	Bred variety (Bv)
62	Xiamao Guozhe	Local variety (Lv)
63	Xiangnan 74-9	Bred variety (Bv)
64	Xiantao Guozhe	Local variety (Lv)
65	Yacheng Qingpi	Local variety (Lv)
66	Yiwu No.25	Bred variety (Bv)
67	Yuanhong 33	Bred variety (Bv)
68	Yunnan Luohanzhe	Local variety (Lv)
69	Zhanjiang Qingpi	Local variety (Lv)
70	Zhongguo No.1	Bred variety (Bv)
