# Supplementary material for: Genetic Diversity Evaluation of 70 Chewing Cane Germplasm Resources Based on Phenotypic Traits
Source: Plants (Basel). 2025 Oct 9;14(19):3111. doi: 10.3390/plants14193111 (PMC12526319; doi:10.3390/plants14193111)
Supplement: Supplementary file 1 [file plants-14-03111-s001.zip › Table S2. Grading criteria for 27 qualitative traits of chewing cane germplasm resources.pdf]

Table S2. Grading criteria for 27 qualitative traits of chewing cane germplasm resources

Type	Phenotypic trait	Grade					
		1	2	3	4	5	6
Stalk-related traits	Aerial root (AR)	None	Less	More			
	Internode form (IF)	Cone	Cylinder	Drum	Slender waist	Curve d	Inverted taper
	Internode arrangement (IA)	Zigzag pattern	Upright pattern				
	Internode color unexposed (ICU)	Red	Yellow	Green	Purple		
	Internode color exposed (ICE)	Red	Yellow	Green	Purple		
	Water crack (WC)	None	Have				
	Cork patch (CP)	None	Have				
	Cork cracks (CC)	None	Have				
	Wax band (WB)	Not obvious	Obvious				
	Internode wax powder (IWP)	None	Thin	Middle	Thick		
	Pipe (Pip)	None	Mild	Middle			
	Pith (Pit)	None	Mild	Middle			
	Growth bands form (GBF)	Unexpansion	Expansion				
	Growth bands color unexposed (GBCU)	Yellow green	Green	Grey orange			
	Growth bands color exposed (GBCE)	Yellow green	Green	Grey orange			
Bud-related traits	Bud form (BFo)	Oval	Triangle	Elliptic	Roundness		
	Bud furrow (BFu)	None	Shallow	Deep			
	Bud placement (BP)	Low	Reach	High			
	No.10 hair group (10HG)	None	Have				
	Bud size (BS)	None	Small	Middle	Big		
	Bud wing size (BWS)	None	Narrow	Wide			
	Lateral budding (LB)	None	Have				
Leaf-related traits	Leaf posture (LP)	Drooping	Erect with carved near tip	Erect			

Leaf color (LC)	Yellow green	Green	Purple	
Leaf sheath phimosis (LSP)	Detached	Easy detach	Hard to detach	
Leaf sheath color (LSC)	Yellow green	Green	Green with purple spots	Purple
No.57 hair group (57HG)	None	Sparse	Thick	
