# Supplementary material for: Genetic Diversity Evaluation of 70 Chewing Cane Germplasm Resources Based on Phenotypic Traits
Source: Plants (Basel). 2025 Oct 9;14(19):3111. doi: 10.3390/plants14193111 (PMC12526319; doi:10.3390/plants14193111)
Supplement: Supplementary file 1 [file plants-14-03111-s001.zip › Table S5. Private alleles in chewing cane across different origins.pdf]

Table S5. Private alleles in chewing cane across different origins

Population	Trait	Grade	Frequency
Bred variety (Bv)	Br	1	0.059
Bred variety (Bv)	CY	9	0.059
Bred variety (Bv)	CY	10	0.059
Bred variety (Bv)	IN	1	0.059
Bred variety (Bv)	LC	3	0.059
Bred variety (Bv)	PH	1	0.176
Bred variety (Bv)	SD	10	0.059
Bred variety (Bv)	SucC	1	0.059
Bred variety (Bv)	WpP	9	0.059
Bred variety (Bv)	WpP	10	0.059
Introduced variety (Iv)	IN	2	0.083
Local variety (Lv)	10HG	2	0.098
Local variety (Lv)	Br	2	0.024
Local variety (Lv)	CY	8	0.024
Local variety (Lv)	GBCU	3	0.122
Local variety (Lv)	IF	5	0.049
Local variety (Lv)	IN	3	0.024
Local variety (Lv)	IN	4	0.146
Local variety (Lv)	PH	9	0.024
Local variety (Lv)	PH	6	0.098
Local variety (Lv)	Pit	2	0.195
Local variety (Lv)	SL	9	0.024
Local variety (Lv)	SL	10	0.049
Local variety (Lv)	SL	1	0.073
Local variety (Lv)	SucC	2	0.024
Local variety (Lv)	WpP	8	0.024
